# Early Infant Male Circumcision in Cameroon and Senegal: Demand, Service Provision, and Cultural Context

**DOI:** 10.9745/GHSP-D-15-00185

**Published:** 2016-07-02

**Authors:** Ernest Kenu, Tin Tin Sint, Claude Kamenga, Rene Ekpini

**Affiliations:** a University of Ghana, School of Public Health, Department of Epidemiology and Disease Control, Accra, Ghana; b United Nations Children’s Fund (UNICEF), New York, NY, USA; c UNICEF, West and Central Africa Regional Office (WCARO), Dakar, Senegal

## Abstract

**Background:**

Male circumcision is almost universal in North and West Africa, and practiced for various reasons. Yet there is little documentation on service delivery, clinical procedures, policies, and programmatic strategies. The United Nations Children’s Fund (UNICEF) commissioned country program reviews in 2014 to shed light on the delivery of male circumcision services for infants in Cameroon and Senegal.

**Methods:**

We conducted a policy desk review, key informant interviews, and focus group discussions at health centers and in communities. Between December 2014 and January 2015, we conducted 21 key informant interviews (13 with regional and district officers, 5 with national officers, and 3 with UNICEF officials) and 36 focus group discussions (6 with men, 6 with women, 12 with adolescent boys, and 12 with service providers). Some of the men and women were parents of the adolescents who participated in the focus group discussions. In the French-speaking areas, the focus group discussions were conducted in French through an accredited translator, audio recorded, and transcribed into English.

**Results:**

All of the facilities we visited in Cameroon and Senegal offer medical male circumcision, with 10 out of 12 performing early infant male circumcision (EIMC) routinely. Neither country has policies, guidelines, or strategies for EIMC. The procedure is done mainly by untrained service providers, with some providers using modern circumcision devices. There are no key messages on EIMC for families; the increasing demand for EIMC is led by the community.

**Conclusion:**

Despite the absence of national policies and strategies, EIMC is routinely offered at all levels of the health care system in Cameroon and Senegal, mainly by untrained service providers. Improving circumcision services will require guidelines for EIMC and improvements in training, equipment, supply chains, recordkeeping, and demand creation.

## Abstract (precis)

Despite the absence of national policies and strategies, early infant male circumcision is routinely offered at all levels of the health care system in Cameroon and Senegal, mainly because of community demand. Improving medical male circumcision will require service guidelines, preservice training, investigation of surgical and nonsurgical devices, supply chains, data collection tools, engaged communities to raise awareness, and communication strategies for men.

## INTRODUCTION

Male circumcision is typically referred to as the complete removal of the foreskin tissue that covers the tip or head of the penis. It is one of the oldest and most common surgical procedures worldwide, and is undertaken for many reasons: religious, cultural, social, and medical. It is widely practiced and almost universal in parts of West Africa, Central Africa, in most Muslim countries, Israel, and the United States.^1^

The hypothesis that male circumcision might protect against HIV infection was first suggested in 1986.^2^ Clinical trials conducted in sub-Saharan Africa revealed that medically performed circumcision is safe and can reduce men’s risk of acquiring HIV infection from heterosexual exposure by about 60%.^3^^-^^5^ In 2007, the World Health Organization (WHO) and the Joint United Nations Programme on HIV/AIDS (UNAIDS) convened a technical consultation to review these new data. The regional consultation in Montreux, Switzerland, recommended male circumcision in countries with high HIV levels, a generalized HIV epidemic, and low prevalence of male circumcision.^6^ The recommendations called for first scaling up services for young and older men who are at high risk of HIV as a priority, and once saturation is reached in these age groups, expanding the services to male infants and young adolescents. Male circumcision within the first 60 days of life, called early infant male circumcision (EIMC), is less costly, less complicated, and less risky than circumcision performed later in life.^7^

EIMC is less costly, less complicated, and less risky than circumcision performed later in life.

Although male circumcision is almost universal and culturally accepted in many countries in West and Central Africa, there is little information on national policies and guidance, service provision, adverse events, training, and demand for the procedure. The United Nations Children’s Fund (UNICEF) commissioned an EIMC program review in 2014 in Cameroon and Senegal to answer questions around 4 areas: service delivery, systems, policy, and monitoring.

### Country Demographics

Cameroon is a bilingual country with defined francophone and anglophone areas. It is a young country with 63% of the urban population and 65% of the rural population under the age of 25 years. Only about two-thirds of women of reproductive age report receiving antenatal care from a skilled service provider. Cameroon has a national HIV prevalence of 4.3% among people ages 15 to 49 years. The average male circumcision rate is 90%, though it ranges from 75% to 100% in various regions. More than 90% of people in urban and rural areas report having no health insurance.^8^

In Senegal, a francophone country, 94% of women of reproductive age report receiving antenatal care from a skilled service provider. The national HIV prevalence is 0.7% of people ages 15 to 49 years, and 97% of men and 95% of women report having heard of HIV. The country has almost universal male circumcision, ranging from 75% to 100% depending on the region. Ninety-eight percent of people do not have health insurance.^9^ Characteristics of the 2 countries are listed in Table 1.

**TABLE 1:** Characteristics of Men and Women Ages 15–49 in Cameroon and Senegal

Characteristics	Cameroon	Senegal
Male circumcision rate	90%	80%
Religion		
Catholic	40%	0%
Protestant	30%	0%
Muslim	20%	95%
Other	10%	5%
HIV prevalence	4.3%	0.7%
Age at sexual debut		
Women	17.1	19.0
Men	18.7	22.7
ANC with skilled attendant (among women)	64%	94%
PNC (among women)	58%	68%
Place of birth of last child		
Public health facility	61%	69%
Home	37%	27%
Multiple sexual partners in the last 12 months		
Women	4%	0.3%
Men	23%	8%
Polygamy (among men)		
Urban	11%	5%
Rural	16%	18%
Total	13%	17%
Knowledge of HIV		
Women	96%	95%
Men	98%	97%
Have had HIV test and know results		
Women	51%	28%
Men	40%	17%
No health insurance coverage		
Women	98%	94%
Men	96%	92%

Abbreviations: ANC, antenatal care; PNC, postnatal care.

Source of data: 2011 Demographic and Health Survey in Cameroon^8^ and a 2010–2011 Demographic and Health Survey – Multiple Indicator Cluster Survey in Senegal.^9^

## METHODS

The review comprised key informant interviews at national, regional, and district levels and with health facility in-charges; health facility assessments; focus group discussions with service providers and men, women, and adolescents, the beneficiaries of EIMC services; and policy framework desk reviews.

### Selection of Review Sites

We selected areas for the reviews based on health indicators and demographic data from a 2011 Demographic and Health Survey (DHS) in Cameroon^8^ and a 2010–2011 DHS-Multiple Indicator Cluster Survey (MICS) in Senegal,^9^ with the aim of achieving a diverse sample of the population and health systems. We took ease of travel and time limitations into account. In Cameroon, we selected the Central, Littoral, and Southwest regions, and in Senegal, we selected the Dakar, Kaffrine, and Tambacounda regions. Within each region, we visited 2 health facilities (1 teaching or regional hospital and 1 health center or health post). We visited 6 facilities in each country (Table 2).

**TABLE 2:** Facilities Selected for EIMC Assessment in Cameroon and Senegal, by Region

Region	Facility
**Cameroon**	
Southwest region (anglophone)	Regional Hospital, Buea
	CMA, Limbe
Littoral region (francophone)	CMA Delange, Edea
	District Hospital, Pouma
Central region, Yaounde (francophone)	Central Hospital, Yaounde
	Hôpital Gynecologie Obstetrique et Pediatrique de Yaounde
**Senegal**	
Western area: Tambacounda	CS Diankhe Makhan
	Centre Hospitalier Regional de Tambacounda
Central area: Kaffrine	CSR Kaffrine
	CS Malem Hodar
Eastern area: Dakar	CS Camberene
	Hôpital Général de Grand Yoff

Abbreviations: CMA, Centre Medical d’Arrondissement (district medical center); CS, centre de santé (health center); CSR, centre de santé regional (regional health center); EIMC, early infant male circumcision.

In Cameroon, 3 facilities were teaching hospitals, 2 were health centers, and 1 was a district hospital. All of the facilities were government owned. In Senegal, 3 of the facilities were health centers, 2 were regional hospitals, and 1 was a teaching hospital. Of the 6 health facilities, the central government owned 4, the local government owned 1, and an NGO owned 1.

### Information Collection

Data collection tools were developed in English, tested in Ghana, and revised based on the field test (see supplementary material). UNICEF had the data collection tools translated into French for the francophone sites. Information on national guidelines, policies, reports, and other related documents on EIMC was collected through (1) key informant interviews and consultative meetings with key stakeholders at national, regional, and district levels and with facility in-charges; (2) facility assessment and focus group discussions with service providers at the health facilities visited; and (3) focus group discussions with mothers of infants who have had circumcision, including pregnant, lactating, and older women; men and young adolescents who were circumcised when they were infants; and community leaders and traditional circumcisers.

For each visit, the team consisted of the lead investigator, the national HIV focal person(s) or representative(s), and the regional director of health services or a representative. An accredited translator accompanied the team in Senegal, whereas in Cameroon, the ministry officials who were part of the team acted as translators where necessary. The team had the questionnaires in both languages at all times.

We conducted 21 key informant interviews and 36 focus group discussions. Of the 21 key informant interviews, 13 were with regional and district officers, 5 with national officers, and 3 with UNICEF officials at the regional and country offices (Table 3). Thirteen of the key informants were men ages 35 to 57. For the 36 focus group discussions, 6 were with men, 6 with women, 12 with adolescent boys, and 12 with service providers (Table 4). Some of the men and women were parents of the adolescents interviewed. A minimum of 3 community focus group discussions were conducted in each of the regions, with each group comprising 8 to 12 participants. One focus group discussion was conducted for women only (including the pregnant, lactating, and older women and women who have had their children circumcised). Another was conducted for men who were community-based resource persons, such as traditional circumcisers, community leaders, and male partners; the last focus group discussion was with adolescent boys who had had EIMC. In the French-speaking areas, the focus group discussions were conducted in French through the accredited translator, audio recorded, and transcribed into English.

We conducted 21 key informant interviews and 36 focus group discussions.

**TABLE 3:** Number of Key Informants, by Country and Affiliation

Cmuntry	National Officers	UNICEF Officials	Regional and District Officers	Total
Cameroon	3	1	7	**11**
Senegal	2	2	6	**10**
**Total**	**5**	**3**	**13**	**21**

Abbreviation: UNICEF, United Nations Children’s Fund.

**TABLE 4:** Number of Focus Group Participants, by Type, Age Group, and Country

	Cameroon	Senegal
Service providers^a^		
Men, 26–57 years	18	16
Women, 24–50 years	32	32
*Subtotal*	*50*	*48*
Community members^b^		
Men, 25–80 years	28	30
Women, 22–52 years	35	32
Adolescents, 12–19 years	33	34
*Subtotal*	*96*	*96*

a 6 focus group discussions with service providers were conducted in each country.

b 12 focus group discussions with community members were conducted in each country.

We conducted a comprehensive assessment of the selected sites using a health facility assessment tool. This tool collects information on the range of services offered, EIMC service provision, availability of policy documents for general service delivery, demand creation, human resource capacity, and quality control measures.

### Information Gathering and Review

Information gathered from the key informant interviews and the responses from the focus group discussions (with adolescent boys, men, women, and service providers) were translated into English, where necessary, and summarized along themes. Data collected from the health facilities and service providers using Data Collection Tool 2 (see supplementary material) were further coded and entered into Microsoft Excel and cleaned before the analysis was done.

Analysis of quantitative data involved summarizing and simple descriptive statistics, such as frequencies and proportions. We processed and summarized the qualitative data to derive the emerging themes, patterns, and key issues. Whenever feasible, we retained verbatim quotes from the respondents to put the findings into context. These findings were checked against the quantitative data from the service delivery points.

### Validation of Findings

We shared key findings at a national validation meeting attended by some participants of focus group discussions, service providers, and policy makers who were interviewed.

### Ethical Consideration

We obtained approval from the ministries of health of Cameroon and Senegal to undertake the assessment. In addition, UNICEF shared the data collection tools in advance for comments and received approval for use before the visits. All participants gave their written consent before the discussion. Adolescent boys who were interviewed gave their assent and their parents gave their written consent.

## RESULTS

### Policies and Guidelines

Both Cameroon and Senegal have no written policy documents to guide the EIMC practice. Many policy documents on HIV services and care of children were available in the 2 countries, but none specifically addresses EIMC or circumcision in general.

In both countries, a number of newborn and child health guidelines and protocols were available in the 12 health facilities. These include: examining a newborn, resuscitation, managing a low-birth-weight baby, guidelines for integrated management of neonatal and childhood illness, maternal health/reproductive health guidelines, guidelines for prevention of mother-to-child transmission of HIV, and child health guidelines. However, there was no guideline for the management of circumcision, though the facilities routinely offer the service. Infection prevention and control supplies such as running water, soap, disinfectant, and goggles were all available.

### Beliefs and Acceptability

All 18 regional, district, and national officers from the 6 regions that we visited (excluding the 3 UNICEF officials in order to focus on the country perspective) indicated that circumcision is almost universally accepted and practiced as part of the culture and for religious beliefs. They accepted medical circumcision due to the evidence of HIV prevention by circumcision. However, 8 of the 18 key informants had concerns with circumcision in young infants. Among the key informants, 3 traditional circumcisers, 3 traditional leaders, 1 health worker circumciser, and 1 urologist opposed circumcision in young infants, and said it should be performed only if the health care delivery system is improved.

In Senegal, the chief of the Layenne community in Dakar, which is predominantly Muslim, said the procedure should be done by the seventh day after birth because it has both religious and medical benefits. He said:

A true Muslim must cut that thing off. It is Arabic, it is not halal; there is no better practice than ours.

A leader in Malem Hodar, a Muslim community in Kaffrine region in Senegal, believes traditional circumcision is a form of education, and if a child is too young, it defeats the purpose. The boy must be old enough to be in “Koranic school,” thus between 12 and 15 years old. He reiterated: “It cannot be done before.”

Meanwhile, a faith healer and circumciser in the same region said the right age at which he circumcises children is between 4 and 5 years. According to him, these children should be able to drink holy water, which he prepares, and have the *grigri* (native form of magic) put on them for 2 years. If the child is less than that age, the circumcision may result in complications.

A Muslim traditional leader in Dianke Makha community in Tambacounda region of Senegal pointed out that their community is not used to early child circumcision. Per their tradition, they wait until the children are 4 or 5 years old and bring a group of them for circumcision. However, the chief of the community said: “Our grandparents taught us to do it this way, but now we can change if we must.”

A leader in the Beninua community in Limbe in Cameroon believes the procedure should be done as early as possible.

We people who stay here do it in the hospitals and health centers on the third day of birth unless there is a problem with the baby, but some people wait for the children to grow small; but from this community, for our tradition we do it fast.

The practice in the francophone region (Littoral and Central) was the opposite of the anglophone region of Cameroon, where they believe the practice should be done between ages 2 and 10 years. It signifies transition from childhood to manhood and at the same time it is a sign of bravery, potency, and an ability to satisfy their future wives; hence it should not be done too early. According to the head teacher in the community and an opinion leader, if the procedure is done too early, it affects the size of the penis:

Eiii [sound of distress], the size of the penis will be small, the man will be weak and cannot make their wives happy, so we do not do it early.

This assertion was not supported by 4 professionals who were interviewed. A urologist who was also a professor at a university said:

Penile size has to do with genetic makeup and other environmental factors as well as use and disuse of the penis.

In general, mothers and their sons in Cameroon were satisfied with the outcome of the circumcision done by both traditional circumcisers and health providers; 1 person noted that her son’s circumcision was not done properly, and she is considering a second circumcision for him. Health facility records showed that acceptance of EIMC was high in the Southwest (anglophone) region of Cameroon (Buea, Tiko, and Limbe). Information from Buea Regional Hospital, for example, showed that 93% (241/259) of infants delivered at the hospital in 2014 were brought back for EIMC, and on average had the intervention done on day 14. Similarly, the Centre Médical d’Arrondissement (CMA) (district medical center) Limbe documented 99% (109/110) EIMC acceptance in 2014.

### Demand for EIMC Service Provision, Advocacy, and Communication

In both Cameroon and Senegal, demand for circumcision services emanates from the community. There are no national communication strategies, mass media campaigns, and information, education, and communication materials (e.g., posters) in the facilities. Community leaders and fathers and mothers of male children regularly ensured that circumcisions were conducted as part of their cultural and religious practices.

In both Cameroon and Senegal, demand for circumcision services emanates from the community.

With the exception of the Urologist Association of Cameroon, which organized a series of lectures and presentations on circumcision,^10^ neither country has developed messages or materials that integrate local issues into explanations of male circumcision and reduced risk of HIV transmission, the healing period, risk of adverse events, who should perform the procedure, where it should be done, at what age, and informed consent issues. Facilities providing circumcision services provide their own form of messages that are appropriate for their setting, such as the medical importance of infant circumcision, the timing of the intervention to be in line with their traditional beliefs, and wound care. The main sources of information about circumcision reported by respondents included traditional leaders, spiritual leaders, and service providers.

### Availability and Accessibility of EIMC Services

The 6 facilities assessed in each country provided a comprehensive set of services (Table 5). In Cameroon, 5 of the 6 facilities provide EIMC and 3 have nutrition services. Of the 5 facilities that provide EIMC services, 3 include EIMC counseling as part of postnatal counseling for mothers who deliver boys. The facilities accepted complications referred from the communities, and most of these cases were seen at the Central Hospital of Yaounde, where there were a number of consultant urologists. In Cameroon, 79% (76/96) of parents indicated that they were able to access EIMC services anywhere they wanted, and that fathers made the decision to have their infant son circumcised. Ten percent (10/96) delayed circumcision due to the cost of the procedure.

**TABLE 5:** Number of Facilities Visited in Cameroon and Senegal Providing Selected Types of Services

Service	Cameroon (N = 6)	Senegal (N = 6)
General OPD services	6	6
Antenatal care	6	6
HIV testing and counseling/PMTCT	6	6
Male circumcision	6	6
EIMC	5	5
EIMC counseling in ANC and postnatal care	3	2
Child health services	6	5
Laboratory services	6	5
Recommended immunization	6	4
Nutrition services	3	3

Abbreviations: ANC, antenatal care; EIMC, early infant male circumcision; OPD, outpatient department; PMTCT, prevention of mother-to-child transmission.

In Senegal, EIMC services were available in all 3 regions visited. Of the 5 health facilities that provide EIMC, only 2 include counseling for mothers who deliver boys. The counseling covered the need to circumcise and who to see for the procedure when the parents are ready. The counseling did not include detailed information on EIMC, where and when it should be done, the clinical procedure, risk of adverse events, and potential benefits.

In both countries, parents bear the cost of the service, either full cost recovery or the subsidized cost. Costs range from 1,000 CFA to 25,000 CFA (US$1.70 to US$42.70) in health facilities, compared with 3,000 CFA to 5,000 CFA (US$5.10 to US$8.50) when performed by traditional circumcisers. In the Southwest region of Cameroon, some facilities operate performance-based funding supported by an NGO, Agence Européene pour le Développement et la Santé (AEDES) and Institut pour la Recherche, le développement Socio-économique et la Communication (IRESCO) (AEDES/IRESCO). The NGO pays the difference between the subsidized fees received from parents and the actual cost of the circumcision. However, in traditional settings, circumcision may be done for free.

In both Cameroon and Senegal, parents bear the cost of the service, either full cost recovery or the subsidized cost.

### Human Resource Capacity and Infrastructure

Although all cadres of health personnel perform circumcision, only urologists and general surgeons are formally trained. Qualified doctors have transferred skills through on-the-job training for operating room nurses and other cadres of workers. These highly motivated and dedicated staff use their own tools most of the time and carry out the service either before or after their normal work schedule. Nevertheless, we noted that the maximum number of staff directly involved in EIMC in each of the facilities was 2.

Most infant circumcisions (90%) were done in the surgical department or any available sterile area within the hospital, including the surgical preparation room. The service providers we interviewed thought that, though circumcision is surgical, it should be housed under reproductive, maternal, newborn, and child health care.

### EIMC Commodities

Cameroon and Senegal do not have national clinical protocols and guidelines that list the medicines, supplies, and equipment needed to deliver all aspects of EIMC services. Most of the commodities, such as scalpel blades, syringes, anesthetic agent, gauze, and suture material, are procured for other services like major surgery and not specifically for circumcision.

In Cameroon, none of the service providers had ever used the reusable Mogen clamp and disposable devices like Plastibell and AccuCirc; instead they use a scalpel and scissors for the circumcision. In contrast, in Senegal, 5 of the facilities visited used the Mogen clamp, and only 1 had a disposable device like Plastibell. From the assessment, 2 of 5 policy makers at the national level and 70% (67/96) of service providers think key circumcision commodities should be in the list of national essential medicines and equipment and in the procurement and distribution systems used by service delivery sites.

### Monitoring and Evaluation and Strategic Information

Monitoring and evaluation officers did not have EIMC data collection and reporting tools in any of the facilities visited. The national monitoring and evaluation tool does not collect data on circumcision. Circumcision services were therefore recorded in different tools available to the service provider. Two of the facilities in Senegal that performed circumcisions were not collecting data on circumcision, and the other 4 that were collecting circumcision data did not record the ages of the infants. Hence, we could not determine the EIMC rate for the facilities visited. The 6 facilities visited in Senegal recorded 6,608 deliveries in 2013.

### Findings From the Focus Group Discussions for Cameroon

All of the 146 participants involved in the community and service provider focus groups in Cameroon were aware of and had knowledge of circumcision. Participants from the Southwest (anglophone) region said that they circumcise their infants between 0 and 60 days of life, which fits the definition of EIMC. Mothers in the anglophone region knew some of the medical benefits of circumcision. Adolescents in the anglophone region of Cameroon indicated that circumcised babies were protected from certain illnesses and diseases, such as urinary tract infection, and the infant may never remember the pain of the procedure. Their parents, however, were not sure of any medical benefit of circumcision.

The participants from the Littoral and Central (francophone) regions said they prefer to circumcise their sons between ages 2 and 10 years. Meanwhile, mothers from the francophone regions reported mainly cultural reasons for circumcision. Sixty-eight percent (24/35) of mothers in the francophone regions prefer circumcision done after age 5 to ensure penile size is not affected. They fear that infant circumcision will cause the penis to become small and interfere with their sons’ ability to satisfy future wives. Similar to the views of francophone women, francophone men prefer to have circumcision at a later age. One of the men said:

Circumcision is like childbirth in women, a warfare where real men are separated from boys.

Adolescent boys in the francophone region enjoy the celebratory feast associated with circumcision and therefore prefer to be circumcised late, which is directly opposite of the views of adolescent boys from the anglophone region. In addition, the francophone adolescent boys said the procedure should be done between ages 5 and 7 years:

You should feel the pain but not cry to show that you are brave, hmmm even remember who cut the thing for you; if you finish, big cock [rooster] be used for party for you to eat all, very, very nice.

Adolescent boys in the francophone region of Cameroon enjoy the celebratory feast associated with circumcision and therefore prefer to be circumcised late, which is opposite of the views of adolescent boys from the anglophone region.

All of the adolescent boys reported that after the procedure is carried out, they really enjoy the biggest rooster prepared for them on that special day. One of the boys said:

If the procedure is done too early, this culture will die.

All parents engaged in the focus groups did not know if service providers had been trained or not. Ninety percent of parents (57/63) had the service provided for their sons by health workers who sometimes provide the service in the homes of their clients.

The following were some of the suggestions from respondents for improvement of EIMC services:

- Education in the various communities about EIMC will allow leaders to start sending the children for circumcision early.
- Traditional circumcisers should be trained with the modern circumcision methods.
- Educational materials need to address the cultural belief that circumcision is an act of manhood that proves bravery and potency.

### Findings From the Focus Group Discussions for Senegal

A total of 96 people were involved in the 12 community focus groups in the 3 regions visited; 3 with men, 3 with women, and 6 with adolescent boys. In addition, 48 service providers were part of 3 focus groups. Most of them affirmed the availability of the EIMC services and their knowledge of the benefits of EIMC, and they were satisfied with the service provision. A participant from the predominantly Muslim Layenne community phrased the benefits as:

The first benefit is, science confirms that a child circumcised as a baby will be protected from certain illnesses and diseases, like phimosis and balanitis. And on a religious level, an adult should not show his private parts, so it is better to get him circumcised at a young age, so that he does not have to show his private parts when he is an adult. Some scientists and doctors also say that the child feels less pain when he is very young.

Other observations made in the focus group discussions were:

- If the procedure is done when the child is a baby, it will be less costly for the parents.
- Without circumcision, one cannot marry, have children, nor get into paradise.

Circumcision was common knowledge to all 144 participants in the community and service provider focus groups. However, 78% (112/144) were not familiar with the term EIMC, except for the communities in Dakar, where EIMC is the norm. Eighty-three percent (80/96) of parents used traditional circumcisers because they consider circumcision to be a religious practice and because it was more cost-effective. Overall, 83% (120/144) of respondents were satisfied with infant circumcision done for their sons or observed by both traditional circumcisers and service providers.

With one exception, none of the service providers and community focus group participants mentioned any serious complications of circumcision. One traditional circumciser mentioned a complication resulting in a “burst” penis during sexual intercourse in a 17-year-old 2 weeks after the circumcision (which he was able to fix).

Other observations from the focus groups:

- Fathers were the main decision makers when it comes to when and where a male child should be circumcised.
- In communities where the practice is carried out as part of religious activity, children are grouped for circumcision and the decision is made by the religious leader of the community. These boys are kept together until wound healing has occurred.
- A minority of men and 1 traditional circumciser believed the age at circumcision has an effect on the size of the penis, which influenced their decision on the timing of the circumcision.

## DISCUSSION

Despite the absence of national policies, strategies, and guidelines on EIMC, it is routinely offered at all levels of the health care system in Cameroon and Senegal, mainly driven by community demand. Across the continuum of care, 10 of the 12 facilities assessed in both countries provide EIMC services.

The decision about when and where to perform the circumcision, and by whom, is made predominantly by fathers and occasionally by community leaders. For religious and cultural reasons, some communities and service providers prefer circumcision to be done between ages 2 and 15 years.

The decision on when and where to perform the circumcision, and by whom, is made predominantly by fathers and occasionally by community leaders.

There is no formal EIMC training for most service providers. The use of modern circumcision devices such as Plastibell and AccuCirc was rare. However, there were not many severe adverse events reported or observed during the assessment. The equally good innovative means of teaching through skills transfer during on-the-job training by qualified doctors for operating room nurses and other cadres of workers served as a stopgap measure for capacity building.

EIMC by service providers is either subsidized or fully paid for by parents. Payments cannot be made through national health insurance, but there is either full cost recovery from or cost sharing with the government or an NGO for some facilities in the Southwest region of Cameroon. Infant circumcisions done for purely traditional reasons by traditional circumcisers are usually done for free, though in some cases parents pay for the service.

## RECOMMENDATIONS

A number of steps could be taken to improve and guide a more systematic provision of EIMC services in Cameroon and Senegal:

- Develop a clear policy and guidelines on provision of medical male circumcision for infants by health cadres.
- Create community engagement to establish a strong community coalition and raise awareness of medical services for infant circumcision.
- Develop communication strategies targeting men and influential members of the community to use trained service providers and/or trained traditional circumcisers for their infant circumcision.
- Develop and include medical male circumcision in preservice training for all health cadres.
- Investigate the potential to use both surgical and nonsurgical devices for EIMC.
- Set up EIMC commodities and supply chains and introduce data collection tools.

These recommendations may also be relevant for other countries where male circumcision is the norm, but where there is no formal system for circumcision in general and EIMC in particular.

## CONCLUSION

EIMC is routinely offered at all levels of service delivery in Cameroon and Senegal mainly due to community demand, albeit in the absence of national policies and guidelines. Improvement of medical EIMC requires the development of guidelines, preservice training, the use of surgical and nonsurgical devices, supply chains, data collection tools, engaged communities to raise awareness, and communication strategies for men.

## Supplementary Material

supplementary material

## Notes

**Peer Reviewed**
