# Supplementary material for: Early Infant Male Circumcision in Cameroon and Senegal: Demand, Service Provision, and Cultural Context
Source: Glob Health Sci Pract. 2016 Jul 2;4(Suppl 1):S18–28. doi: 10.9745/GHSP-D-15-00185 (PMC4944576; doi:10.9745/GHSP-D-15-00185)
Supplement: supplementary material [file GHSP-D-15-00185_index.html]

Supplement to Early Infant Male Circumcision in Cameroon and Senegal: Demand, Service Provision, and Cultural Context | Global Health: Science and Practice

## GHSP-D-15-00185 Supplementary Material

Kenu et al. doi: 10.9745/GHSP-D-15-00185

- supplementary material - Kenu et al. doi: 10.9745/GHSP-D-15-00185
