# Supplementary material for: Early Infant Male Circumcision in Cameroon and Senegal: Demand, Service Provision, and Cultural Context
Source: Glob Health Sci Pract. 2016 Jul 2;4(Suppl 1):S18–28. doi: 10.9745/GHSP-D-15-00185 (PMC4944576; doi:10.9745/GHSP-D-15-00185)
Supplement: supplementary material [file 15-00185-Kenu-Supplementary-material.pdf]

Data Collection Tools

Tool 1: Key Informants

1.1 EIMC Focal person/ Managers/ Officers/

1. Guidelines and policies

What are the EIMC specific guidelines and policies available in this country? Kindly share the document with the consultant/team.

- What are the guiding principles within the guideline?
- How does the document define EIMC?
- Are EIMC targets specified?
- How is the issue of informed consent addressed?
- Are there laws governing EIMC?
- Where is EIMC situated in the service delivery system (HIV program/PMTCT or MNCH etc)? Is there a collaboration between the HIV and MNCH community? Who takes the lead?
- How were these guidelines/ national strategy created? Who took part in this/ which stakeholders were engaged?
- Which partners/organizations play main roles in implementing EIMC on the national level? Which agency is the lead or focal point?
- What research was done (feasibility, pilots) prior to the creation of the national policy or guidelines?

2. Beliefs and Acceptability

- Effective communication and demand creation
- Concerns about safety and quality
- Culture – are there specific tribes or ethnic groups that are resistant to accepting EIMC?
- Religion- is it having an effect on acceptability?
- What are the current social and behavioural motivators and barriers to EIMC uptake in each geographic area surveyed? Do these seem to vary by religion/ethnic majority/other factor?
- How common is infant circumcision provided by traditional practitioners at the community level? If so, has dialogue between health programmes and chiefs/elders begun in order to shift demand from traditional to medical circumcision?
- Who is generally the primary decision maker for an infant in regards to EIMC? What are main factors in the decision making process?
- What messages are most effective in conveying the risks and benefits of EIMC to parents and caregivers?
- How widely accepted/requested is EIMC? Do service providers recommend it, and if so at what point of care?

3. Services and Human resource

- What are the potential access points for EIMC services in your country? Which of them are currently being used?

- Are there opportunities for education on EIMC at the service delivery points and communities
- ?
- Who are really carrying out EIMC in this country? Kindly mention? Where do they carry out the services
- Do you know of any adverse event following EIMC? What adverse even reporting mechanisms are available at the community and/or facility level?
- Which cadres of health care workers are currently performing EIMC? Have there been discussions around task-shifting?
- What specific trainings are provided on EIMC for service providers? – Can you describe the trainings (duration, frequency, what is covered, who attends)? Are there practicals during the training? Are refresher trainings provided?
- What training manuals are used? Make note of who created the manual: MOH, technical working group, UN agency, etc.
- How frequently does EIMC supply not meet demand (ex. EIMC requested by caregiver but services procedure isn't available)?

4. Infrastructure and supply chain

- What were the main feasibility assessment done for the facilities providing EIMC services?
- Space, equipment and supplies needed
- Devices and clinical protocols used (Reusable device- Mogen clamp or disposable device – Plastibel or AccuCirc)
- How do you get your equipment and supplies?
- What supply chain challenges you can identify? Other general barriers to providing EIMC at the facility level?

5. Funding and Political Support

- What funding is allocated for EIMC in the national budget?
- What is the source of funding for EIMC? Out of pocket or government/donors?
- National Health insurance (and is EIMC included in this insurance plan)?
- Cost to patient for the service?Does EIMC have a political support? How?

6. **What were the main achievements (positive, tangible, attractive and innovative results) for the EIMC for the past 2 years? Aspects to consider include**:

- *Management and coordination of the EIMC (including the funding: sources, levels, trend sustainability strategies)*
- *Uptake of EIMC services and its link to other services thus integration of services (with the other HIV services; with other related programs)*
- *Resources for EIMC:: human resources for health; medicines, infrastructure, equipment and supplies*
- *Advocacy, community education and social mobilisation (includes the involvement of social groups and traditional leaders)*
- *Program monitoring and utilisation of strategic information at all levels*

7. Give a few examples of what you consider "best-practices" for EIMC.

8. Describe the main weaknesses /challenges for EIMC service in the country. (Aspects to consider are the same as above)

9. Monitoring and Evaluation

Is the M&E system addressing the data collection and reporting on EIMC? Consider the following?

- *Integration with HMIS*
- *Data collection tools (registers, cards, reporting forms) availability, accessibility, completeness and accuracy*
- *Management and use of data at all levels*
- *Data base and reporting system;*
- *Adverse events*
- *Operational research on EIMC*

Tool 2: HEALTH FACILITY TOOL

0.1 Date: (dd/mm) |____|____|____|____/2014

0.2 Interviewer Name: ________________________________ **Signature:** ___________________________

0.3 Supervisor Name:________________________________ **Signature:**___________________________

0.4 Health Facility Name: ………………………… **0.5 Health Facility ID:** |____|____| 0.6 Urban or Rural

SECTION 1: HEALTH FACILITY INFORMATION

**1.1**	Type of health facility<br>*Please circle*	1 = Teaching hospital<br>2 = Regional hospital<br>3 = Other hospital<br>4 = Polyclinic	5 = Health Centre<br>6 = Clinic<br>7 = Maternity home<br>8 = CHPS compound
**1.2**	Managing authority<br>*Please circle*	1 = Government<br>2 = Non-governmental organisation (NGO)<br>3 = Faith Based Organisation (FBO)/CHAG<br>4 = Private<br>5 = Other (*specify):*	
**1.3**	Designation of main respondent	1 = Doctor<br>2 = Health Officer<br>3 = Nurse<br>4=Public Health Nurse	5 = Midwife<br>6 = Medical Assistant<br>7 = Health Care Assistants<br>8=Other *(specify):*
**1.4**	Please mention the services provided at this health facility<br>*Circle all those that apply.*	1 = General OPD services<br>2 = Antenatal care<br>3 = Family Planning<br>4 = Maternity/ delivery<br>5 = HCT/ PMTCT	6 = Child health care<br>7 =EIMC (infant circumcision)<br>8 = Nutrition services<br>9 = Laboratory services<br>10 = In-patient services<br>11 = Other *Specify:*
**1.5**	Please tell me about the staffing level at this health facility<br>*Record the number of providers under each cadre.*	1 = Doctors _____<br>2 = House Officers ____<br>3 = Nurses____<br>4 = Midwives ____	5 = Laboratory staff ____<br>6 = Pharmacy staff ____<br>7 = Nursing Aide ____<br>8 = Other *Specify:*____
**1.6**	Please tell me about the number of staff trained in Early Infant Male Circumcision in this facility	1 = Doctors _____<br>2 = House Officers ____	5 = Laboratory staff ____<br>6 = Pharmacy staff ____

	Record the number of providers under each cadre.	3 = Nurses____<br>4 = Midwives ____	7 = Nursing Aide ____<br>8 = Other *Specify*:_____
**1.7**	Is there a trained provider assigned to and present at the health facility at all times (24 hours a day) for EIMC? *Please ask to see a duty schedule for the 24 hour staff coverage*	1 = YES, and the duty schedule observed<br>2 = YES, but no duty schedule seen<br>3 = NO 24 hour onsite staff<br>4 = NO, services provided 8am to 5pm	
**1.8**	Which of the following infection prevention and control facilities were available at the health unit? *Please circle all that apply.*	1 = Water<br>2 = Running water<br>3 = Soap<br>4 = Disinfectant<br>5 = Goggles<br>6 = None	
**1.9**	What type of devices do you use for EIMC at this facility?	1= Reusable device- Mogen clamp<br>2= Disposable device – Plastibel<br>3= Disposable device -- AccuCirc	
**1.10**	Which of the following records are available at this health facility? *Please circle all that apply.*	1 = Discharge forms<br>2 = ANC cards<br>3 = Partograph<br>4 = Death Certificate forms<br>5 = Perinatal audit forms<br>6 = PNC registers	7 = Child Health Cards<br>8 = Clinical case notes<br>9 = Referral forms<br>10 = Birth registers<br>11 = ANC registers<br>12 =other_______________
**1.11**	In which of the following records at this health, do service providers record EIMC	1 = Discharge forms<br>2 = ANC cards<br>3 = Partograph<br>4 = Death Certificate forms<br>5 = Perinatal audit forms<br>6 = PNC registers	7 = Child Health Cards<br>8 = Clinical case notes<br>9 = Referral forms<br>10 = Birth registers<br>11 = ANC registers<br>12 =other_______________
**1.12**	Which of the following guidelines and protocols were available at the health facility? *Please circle all that apply.*	1= Examining a newborn<br>2 = Resuscitation<br>3 = Managing a low birth weight baby<br>4 = IMNCI guidelines<br>5 = Maternal health/ reproductive health guidelines	

		6 = Child health guidelines<br>7 = PMTCT guidelines<br>8 =Guidelines on EIMC/ Policy document on EIMC<br>10 =Others_______________
**1.13**	How many deliveries were done in this facility in 2013	_____________________
**1.14**	How many of the babies were males	
**1.15**	How many of the male infants were circumcised	
**1.16**	Does your facility receive or accept referrals from the communities for EIMC	1= Yes<br>2= No
**1.17**	Does your facility accept complicated circumcision cases from traditionalist who do circumcision?	1= Yes<br>2= No

SECTION 2: NEBORN, INFANT & YOUNG CHILD HEALTH SERVICES

**2.1**	Which of the following immunisations for children are available at this health facility? *Please circle all that apply*	1 = Polio<br>2 = BCG<br>3 = DPT –HB+Hib<br>4 = Measles<br>5 = Rubella<br>6 = Rota Virus vaccine<br>7 = Other *Specify:*		
**2.2**	Is EIMC service offered in this facility?	1=Yes<br>2= No		
**2.6**	Where exactly is the service situated?	1= MNCH<br>2= Surgical Department<br>3=Others specify		
**2.7**	Please observe if the following activities are being conducted routinely. If not seen, please ask. *Please circle the appropriate one.*	OBSERVED	REPORTED, NOT SEEN	NOT DONE ROUTINELY
**A**	Weighing of the child	1	2	3

**B**	Plotting child's weight on the Child Health Card	1	2	3
**C**	Assessing if the infant was exposed to HIV	1	2	3
**D**	EIMC offered	1	2	3

SECTION 3: ANTENATAL CARE SERVICES

**3.1**	Are antenatal care services offered in the health facility on every day that outpatient consultations are provided? *Please circle*	1 = YES, all days<br>2 = YES, some days<br>3 = NO<br>4 = DON'T KNOW		
**3.2**	Are pregnant women offered counselling during ANC visits?<br>*Please circle the appropriate response.*	1 = Yes<br>2 = No		
**3.3**	If Yes, Does the counselling include EIMC?<br>*Please circle*	1 = Yes<br>2 = No		
**3.4**	Counselling on EIMC include the following, Please circle all that apply?	1 = What is EIMC<br>2 = Where it should be done<br>3 = Clinical procedure<br>4 = Human resource (Person to do it)<br>5 = Adverse events<br>6 = Potential benefits		
**3.4**	Which of the following services are routinely offered to antenatal clients at this health facility? *Please circle*	ROUTINELY TO ALL	NOT OFFERED	DON'T KNOW
**A**	Counselling about family planning/ child spacing	1	2	3
**B**	Provider-initiated testing and counselling (PITC)	1	2	3
**C**	EIMC counselling services	1	2	3
**D**	Infant feeding counselling	1	2	3
**E**	Nutrition counselling	1	2	3
**F**	HIV testing for male partners (within context of PMTCT)	1	2	3

Thank you very much!

Tool 3: Focus Group Discussion

Targets:

1) *Pregnant and lactating women who have accessed ANC/Post natal women (Not more than 8)*
2) *Village Health Workers, Community leaders (traditional circumcisors)*
3) *Male partners of pregnant and lactating women*
4) *Health workers from hospitals and clinics (Private and Public)*

Questions

1. What do you know about early infant male circumcision?
   - *Benefits*
   - *Availability*
   - *Accessibility*
   - *Factors that hinder utilisation*
   - *Suggestions for improvement*
2. What do you have to say about its
   - *Acceptability- Is it accepted in the general community? Do you want/expect to circumcise your sons?*
   - *Feasibility- how easy is it to access?*
   - *Safety?*
   - *Ethical concerns?*
   - *Policy and strategic plan?*
   - *Key implementers*
   - *Devices/Tools used for EIMC and why?*
   - *Current implementation- How is EIMC currently been done?*
   - *Registers used, data collection and record keeping*
   - *Financing*
3. What are your experiences with EIMC? Or what you have heard about people's experiences from the EIMC services?
   - *Satisfactory experiences*
   - *dissatisfaction with services*
   - *Delays and spending longer than usual before accessing services*
   - *Negative attitudes and rudeness of service providers*
   - *Stigma related issues*
   - *Discrimination at the service delivery points*
   - *Suggestions for improvement*
4. What communication and community related interventions are you aware of about EIMC?
   - *Sources of information*
   - *Clarity, relevance, adequacy and appropriateness of messages*
   - *Existing community support mechanisms and groups*
   - *Suggestions for improvement*
5. What can you say about male involvement in EIMC services?
   - *Knowledge about EIMC services*
   - *Participation by men in EIMC*
   - *Factors that hinder male participation*
   - *Suggestions for improvement /addressing the bottlenecks*
